# MicroRNA-21 promotes the ovarian teratocarcinoma PA1 cell line by sustaining cancer stem/progenitor populations in vitro

**DOI:** 10.1186/scrt247

**Published:** 2013-07-26

**Authors:** Wei-Min Chung, Wei-Chun Chang, Lumin Chen, Ying-Yi Chang, Chih-Rong Shyr, Yao-Ching Hung, Wen-Lung Ma

**Affiliations:** 1 Graduate Institution of Clinical Medical Science, School of Medicine, China Medical University, Taichung 404, Taiwan; 2 Department of OBS & GYN, Sex Hormone Research Center, China Medical University Hospital, Taichung 404, Taiwan; 3 Department of OBS & GYN, BenQ Medical Center, Suzhou, Zhejiang 215000, People’s Republic of China; 4 Department of Medical Technology, China Medical University, Taichung 404, Taiwan

**Keywords:** microRNA 21, Ovarian teratocarcinoma, Cancer stem/progenitor cells, CD133

## Abstract

**Introduction:**

Resistance of cancer stem/progenitor cells (CSPCs) to chemotherapy can lead to cancer relapse. Ovarian teratocarcinoma (OVTC) arises from germ cells and comprises pluripotent cells that can be used to study cancer cell stemness. In this study, we evaluated whether microRNA-21 (miR-21) promotes ovarian teratocarcinoma by maintaining cancer stem/progenitor populations.

**Methods:**

The lentiviral delivery system was used to upregulate or to suppress the expression of miR-21 in the human ovarian teratocarcinoma cell line PA1 and cell growth assays were used to monitor the expression of miR-21 at different time points. Antibodies directed toward CD133, a stem cell marker, were used to identify CSPCs in the PA1 cell population, and the level of miR-21 expression was determined in enriched CSPCs. Stem cell functional assays (sphere assay and assays for CD133 expression) were used to assess the effects of miR-21 on progression of the CD133+ population.

**Results:**

Knockdown of miR-21 in PA1 cells attenuated growth of PA1 cells whereas overexpression of miR-21 promoted cell growth. Moreover, knockdown of miR-21 resulted in a marked reduction in the CD133+ population and sphere formation of CSPCs. In contrast, overexpression of miR-21 resulted in a marked increase in the population of CD133+ cells as well as sphere formation of CSPCs.

**Conclusions:**

MicroRNA-21 plays a significant role in cancer growth by regulating stemness in cancer cells.

## Introduction

MicroRNAs (miRs) are small non-coding RNAs (22 to 24 nucleotides in length) that negatively regulate post-transcription by binding to the 3′UTR of target messenger RNA [1-4]. Studies have shown that miR-21 functions as an onco-miR by regulating tumorigensis and tumor progression [5-7] and has been found to be frequently up-regulated in cancer stem/progenitor cells (CSPCs) [8-11]. Several studies have demonstrated that knockdown of miR21 inhibits cell proliferation, migration and tumor growth in breast [12-14] and ovarian cancers [15,16]. Furthermore, some reports have shown that up-regulation of miR-21 maintains self-renewal and pluripotency of CSPCs and also regulates epithelial-mesenchymal transition (EMT) in breast cancer stem-like cells [17,18]. However, it is still unclear whether miR-21 promotes cancer by maintaining the pluripotency of stem or progenitor cells.

Ovarian cancer can be grouped by cellular origin, including epithelial cells (ovarian carcinoma), stromal cells (ovarian adenoma) and germ cells (ovarian teratoma and teratocarcinoma (OVTC)) [19,20]. OVTC is a rare, malignant neoplasm consisting of elements of teratoma with those of embryonal carcinoma or choriocarcinoma [21-25]. OVTC is caused by the abnormal development of pluripotent germ and embryonic cells, making it a good model for studying the behavior of CSPCs.

CSPCs are thought to be a confounding factor for tumor recurrence and chemoresistance because of their capacity for unlimited self-renew and differentiation [19,26-29]. Studies have shown that several glycoproteins, namely CD133, CD117, CD24 and CD44, as well as the transcription factors OCT-4 and Nanog are markers of CSPCs [30-34]. CD133 is abundantly expressed in the human OVTC cell line PA1 and is frequently used to enrich CSPCs in studies of cancer stem cell characteristics. In addition to measuring CSPCs markers, tumor sphere formation is also used to detect and enrich CSPCs [19,27].

Recently, lentiviral-based miRNAs delivery systems (antisense-miRNAs to knockdown, or pre-miRNAs to overexpress) were developed to manipulate miRs expressions in cells. By introducing MiRZip-21 microRNA (knockdown) and pre-miR-21 (overexpression), we established a durable and effective miR-21 manipulation method [35,36]. In this study, lentiviral-based miR-21 modulators were used to examine the role of miR-21 in OVTC cells.

## Methods

### Cell culture

The human OVTC cell line PA1 and the human embryonic kidney cell line HEK293T were cultured in ((D)MEM) (Gibco, USA) with 10% FCS and 1% penicillin/streptomycin (Gibco, USA). PA1 cells were provided courtesy of >Dr. Min-Chie Hung (MD Anderson, Houston, TX, USA) and HEK293T cells were obtained from Dr. Yuh-Pyng Sher (Center of Molecular Medicine, China Medical University Hospital, Taichung, Taiwan). The cell lines were maintained at 37°C in a humidified atmosphere of 5% CO_2_.

### Western blotting assay

Protein extraction and the immunoblot assay were performed as previously described [37]. Briefly, cells were washed with 1 x PBS and resolved in RIPA buffer (100 mM Tris, 5 mM ethylenediaminetetraacetic acid (EDTA), 5% NP40; pH8.0) with protease inhibitors (1 mM phenylmethyl sulfonyl fluoride, 1 μg/ml aprotinin, 1 μg/ml leupeptin). Proteins were resolved by SDS-PAGE and then transferred to polyvinylidene difluoride (PVDF) membranes. Blocking of non-specific binding was accomplished by adding 5% non-fat milk. After application of primary antibodies, secondary antibodies were applied (1:3,000, horseradish peroxidase (HRP)-goat-anti-mouse and HRP-goat-anti-rabbit) for one hour at room temperature. Signals were enhanced using an enhanced chemiluminescence kit (Millipore, Billerica, Massachusetts 01821, USA) and detected by ChemiDoc ™ XRS + (BioRad, Hercules, CA 94547).

### Transfection and infection procedure

Basically, the lentiviral production and infection procedures were carried out as reported previously [38]. Briefly, cells were transfected with the following lentivirus plasmids: psPAX2 packaging plasmid, pMD2G envelope plasmid (Addgene, Cambridge MA 02139) [37,38], miRZip-21 [35] (anti-miR-21 microRNA construct; System Biosciences, SBI, Johnstown, PA 15901, USA), and hsa-miR21 [36] (Human Lentiviral Primary microRNA Over-Expression Construct in pMIRNA1; System Biosciences, SBI). Both lentiviral plasmids carried the GFP gene and were co-transfected with psPAX2 and pMD2G into HEK293T cells at a ratio of 3:2:4 by lipofectamine2000 (Invitrogen, USA) as per the manufacturer’s instructions. After six hours, the medium was replaced with fresh (D)MEM/10% fetal bovine serum (FBS) medium and cells were maintained at 37°C in a humidified incubator in an atmosphere of 5% CO_2_ for 48 hours. Medium containing virus was collected by centrifugation and filtered through a 0.45 μm filter. Medium containing 0.8 mg/ml polybrene (Sigma-Aldrich, USA) was then added to culture dishes containing 10^6^ PA1 cells. After 16 hours of infection, the medium containing virus was replaced with fresh (D)MEM/10% FBS medium and cells were maintained at 37°C in a humidified incubator in an atmosphere of 5% CO_2_ for 48 hours. Infected cells were then collected and analyzed. The GFP+ cells were measured by flow cytometry (BD LSR II Flow Cytometry) to determine infection efficiencies. GFP + cells with infection efficiencies greater than 85% were subjected to the following experiments (data not shown).

### Total RNA isolation and cDNA synthesis

RNA was extracted from PA1 cells as reported previously [37]. Briefly, cells that had reached approximately 80% to 90% confluence in 100-mm dishes were lysed with 1 ml Trizol (Invitrogen). Phenol/chloroform was then added and RNA-rich layers were separated by centrifugation. Soluble RNA was precipitated with 2-propanol. RNA was then rinsed with 75% ethanol and dissolved in RNase-free water. For first-strand cDNA synthesis, 5 μg of total RNA was used to perform reverse transcription PCR by the PrimeScript™ RT reagent kit (Takara Bio Inc., Tokyo, Japan). MiRNA cDNA was synthesized using the Mir-X™ miRNA First-Strand synthesis Kit (Clontech, Mountain View, CA 94043, USA). cDNA and miRNA were synthesized according to the manufacturer’s instructions.

### Quantitative real-time PCR analysis

A real-time detection system (Bio-Rad Laboratories, Inc., Hercules, CA, USA) and the KAPA™ SYBR FAST One-Step qRT-PCR Kit (Kapa Biosystems, Woburn, MA, USA) were used according to the manufacturers’ instructions. Relative gene expression was determined by normalizing the expression level of the target gene to the expression level of housekeeping genes (U6 or actin). Threshold value (Ct) dynamics were used (2^-ΔΔCt^) for quantitation of gene expression. The qRT-PCR primer sequences were as follows: CD133 forward 5′- TCT CTA TGT GGT ACA GCC G-3′, reverse 5′- TGA TCC GGG TTC TTA CCT G-3′; CD24 forward 5′- TTT ACA ACT GCC TCG ACA CAC ATA A-3′, reverse 5′- CCC ATG TAG TTT TCT AAA GAT GGA A-3′; CD44 forward 5′- GAC CTC TGC AAG GCT TTC AA-3′, reverse 5′- TCC GAT GCT CAG AGC TTT CTC-3′; CD117 forward 5′- CAA GGA AGG TTT CCG AAT GC-3′, reverse 5′- CCA GCA GGT CTT CAT GAT GT-3′; Nanog forward 5′- GGG CCT GAA GAA AAC TAT CCA TCC-3′, reverse 5′- TGC TAT TCT TCG GCC AGT TGT TTT-3′; OCT-4 forward 5′- GGC CCG AAA GAG AAA GCG AAC C-3′, reverse 5′- ACC CAG CAG CCT CAA AAT CCT CTC-3′; SCF forward 5′- ACT GAC TCT GGA ATC TTT CTC AGG-3′, reverse 5′- GAT GTT TTG CCA AGT CAT TGT TGG-3′; miR-21 5′- TAGCTTATCAGACTGATGTTGA-3′.

### Cell growth analysis

The WST-1 assay (Roche, Indianapolis, Indiana 46250, USA) [39] was used to assess cell growth. Briefly, 10^3^ cells/100 μl/well were seeded in 96-well plates with (D)MEM in 10% FBS and were incubated for the designated time periods (one, two, four, six and eight days). Then 10 μl of WST-1 solution was added to each well and cells were allowed to incubate at 37°C in an incubator for one hour. Cell viability was quantified by colorimetric detection in an ELISA plate reader (Beckman Coulter Paradigm™ Detection Platform, USA) at an absorbance of 450 nm and 690 nm to generate an optical density (OD) proportional to the relative abundance of live cells in the given wells.

### Verification and sorting of CD133+ cells

Cells were detached with 1% trypsin/EDTA and cell membrane non-specific binding to antibodies was blocked by 5% BSA for 30 minutes at room temperature. Cells were then incubated with antibody CD133- allophycocyanin (APC) (magnetic cell separation (MACS), Miltenyi Biotec, Auburn, CA 95602) for 30 minutes and then subjected to flow cytometry (fluorescence-activated cell sorting (FACS), BD FACSAria, USA) analysis. The CD133+/− cells were collected by a cell sorter (BD FACSAria).

### Sorting of CD133+ and CD133– populations

A CD133 MicroBead kit (Miltenyi Biotec) was used to sort PA1 CD133+ cells according to the manufacturer’s instructions. Briefly, 10^8^ cells were washed, resuspended in 300 μl of buffer (1 X PBS/ pH 7.2, 0.5% BSA/2 mM EDTA), with 100 μl FcR Blocking Reagent and 100 μl of CD133 MicroBeads, mixed well, and then incubated for 30 minutes at 2°C to 8°C. The labeled cells were then washed, resuspended in 500 μl buffer and applied on a MACS Column of MiniMACS separation kit to collect the unlabeled negative fraction (that is, CD133– cells). Finally, we moved the column onto collection tubes to collect labeled cells (that is, CD133+ cells).

### Sphere formation assay

Cells were collected and washed to remove serum and then suspended in serum-free (D)MEM supplemented with 20 ng/ml human recombinant epidermal growth factor (hrEGF), 10 ng/ml human recombinant basic fibroblast growth factor (hrbFGF), 5 μg/mL insulin and 0.4% BSA (Sigma). The cells were subsequently cultured in ultra low attachment 6-well plates (Corning Inc., Corning, NY, USA) at a density of less than 5,000 cells/well for 14 days. Spheres were observed under a microscope and images were photographed under a phase contrast fluorescence microscope (Nikon, ECLIPSE 80i).

### Statistical analysis

Statistical analyses were performed using the Student’s t-test. All experiments were repeated at least three times, and *P*-values less than 0.05 were considered to indicate statistical significance.

## Results

### MiR-21 is essential for PA1 cell growth

**Figure 1:**
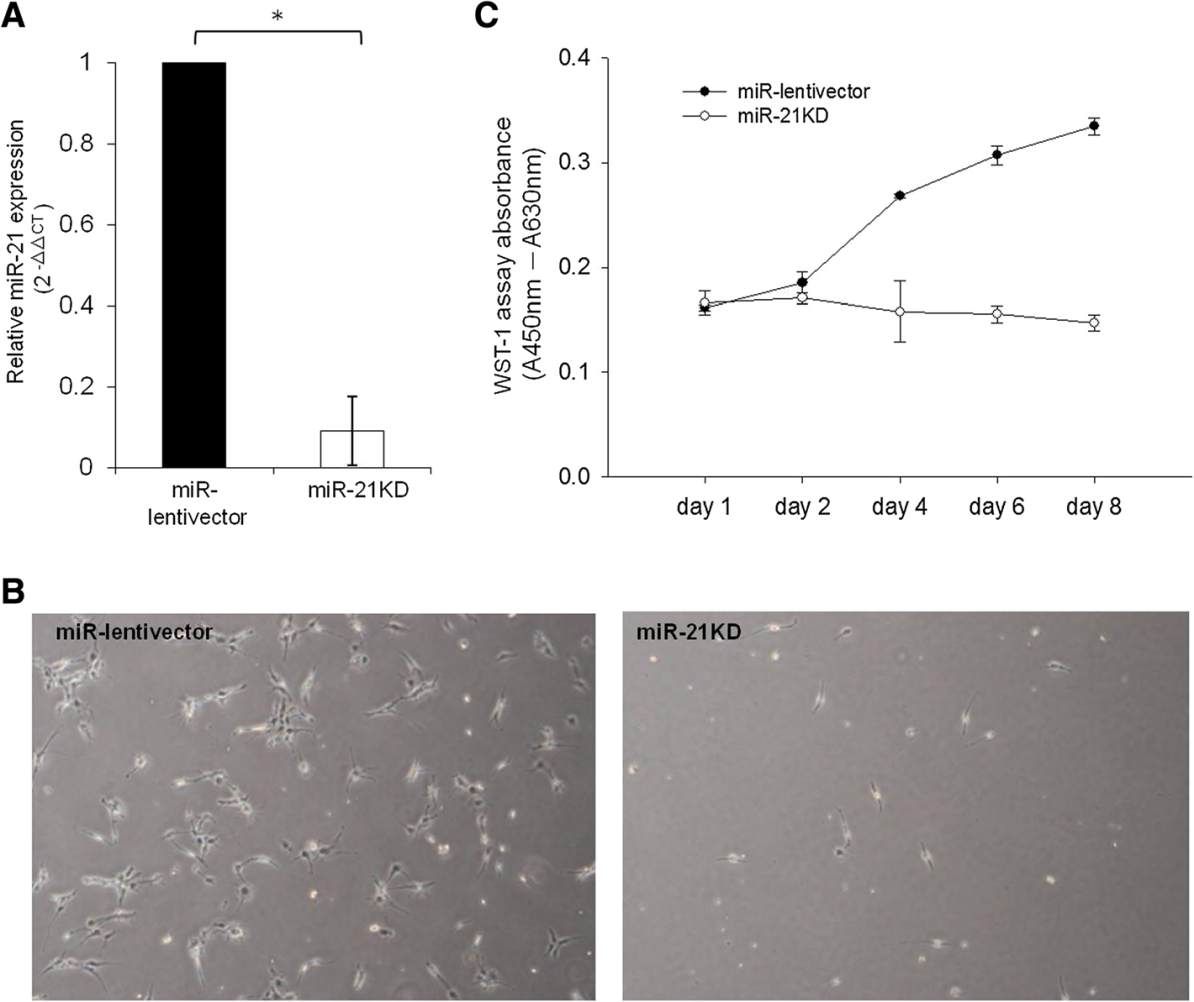
**Knockdown of miR-21 inhibits PA1 cell growth.** MiR-21 expression was significantly lower in PA1 cells infected with anti-miR-21 (miR-21 KD) than in cells infected with control (miR-lentivector) constructs. Relative expression of miR-21 was detected with quantitative real-time PCR and the relative amount of miR-21 is presented as the values of 2^-ΔΔCt^**(A)**. Cell morphology and confluence of vector- or anti-miR-21-infected PA1 cells. Images were photographed on the sixth day of culture using a phase contrast fluorescence microscope (40×) **(B)**. Cell growth WST-1 assays were performed at the indicated time points (one, two, four, six and eight days) and are shown on the X-axis. The Y-axis indicates the absolute absorbance (Abs.) values (Abs. at 450 nm deducted from Abs. at 630 nm background readings). The results shown are from three reproducible experiments **(C)**. ^*^ indicates significance at *P* values less than 0.05. miR, microRNA.

Knockdown of miR-21 has been shown to inhibit cell growth in ovarian cancer *in vitro *[15,16]. To assess whether miR-21 affects cell growth in OVTC, we used the lentiviral delivery system to optimize miRZip-21 constructs. We found that miRZip-21 reduced miR-21 expression levels by 85% in PA1 cells (Figure 1A). We measured the effects of miR-21 knockdown on PA1 cell growth at days one, two, four, six and eight and found that cell numbers had diminished at day six of culture (Figure 1B). As seen in Figure 1C, cell growth was abolished by miR-21 knockdown during all measurement time points. These data suggest that miR-21 is essential for OVTC PA1 cell growth, a finding consistent with that reported in previous studies [12-16].

### Exogenous delivery of miR-21 promotes PA1 cell growth

**Figure 2:**
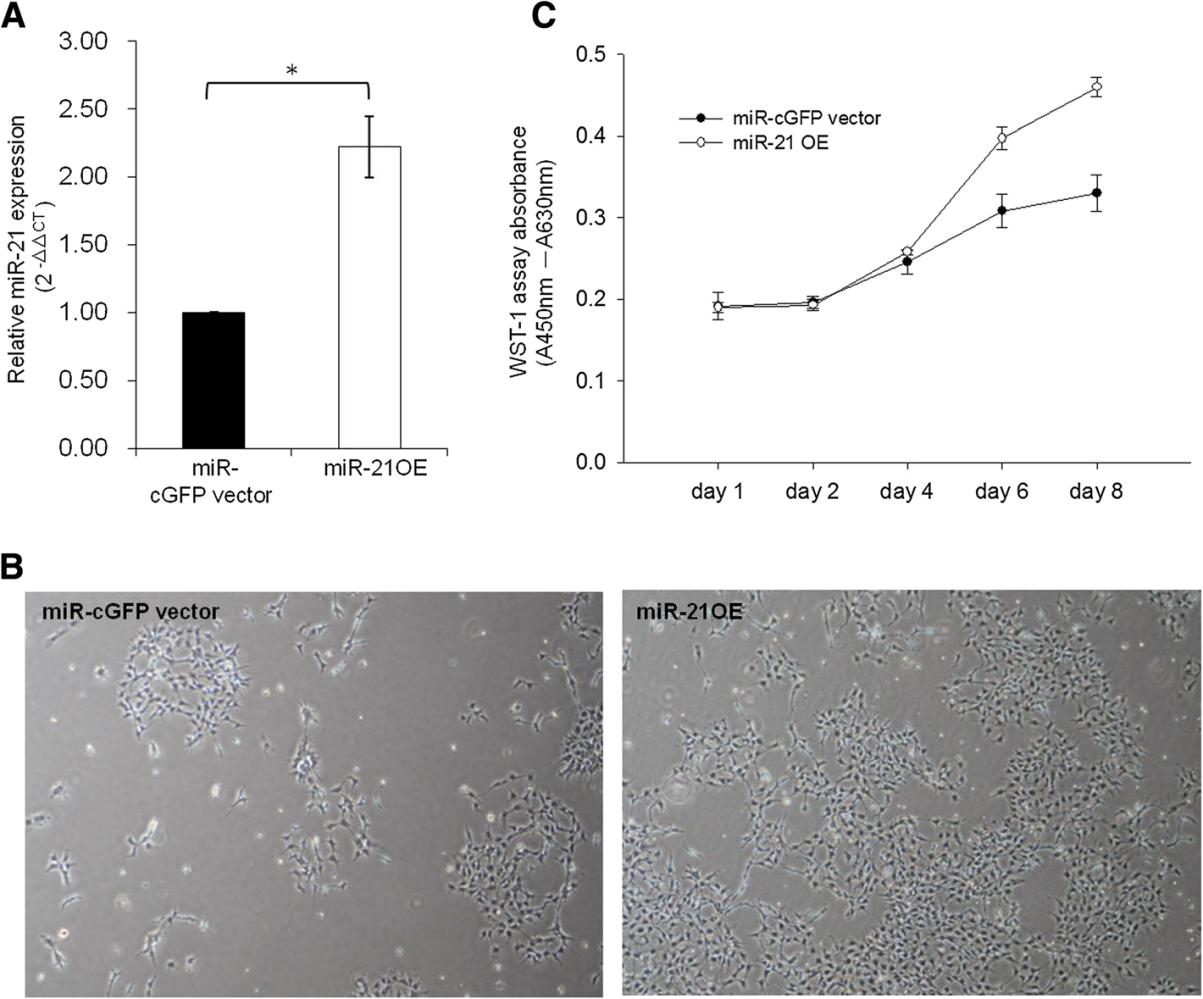
**Overexpression of miR-21 promotes PA1 cell growth.** MiR-21 expression was significantly higher in PA1 cells infected with pre-miR-21 (miR-21 OE) than in cells infected with control (miR-cGFP vector) constructs. Relative expression of miR-21 was detected with quantitative real-time PCR and the relative amount of miR-21 is presented as the value of 2^-ΔΔCt^**(A)**. Cell morphology and confluence of vector- or pre-miR-21-infected PA1 cells. Images were photographed on the fourth day of culture using a phase contrast fluorescence microscope (40×) **(B)**. Cell growth WST-1 assays were performed at the indicated time points (one, two, four, six and eight days) and are shown on the X-axis. The Y-axis indicates the absorbance (Abs.) values (Abs. at 450 nm deducted from Abs. at 630 nm background readings). The results shown are from three reproducible experiments **(C)**. ^*^ indicates significance at *P* values less than 0.05. miR, microRNA; OE: overexpression.

In order to confirm that miR-21 promotes cell growth, hsa-miR21 plasmids (miR-21 overexpression constructs) were introduced to overexpress miR-21 in PA1 cells. In our delivery system, miR-21 expression levels were approximately twice as great as those in the vector control (Figure 2A). As expected, PA1 cells treated with pre-miR-21 had higher growth capacity compared to vector control delivery at day four (Figure 2B). As seen in Figure 2C, cell growth was enhanced by miR-21 overexpression during all measurement time points. This result is consistent with the data presented in Figure 1. The results indicate that miR-21 is essential for PA1 cell growth.

### MiR-21 is up-regulated in PA1 CD133+ cells

**Figure 3:**
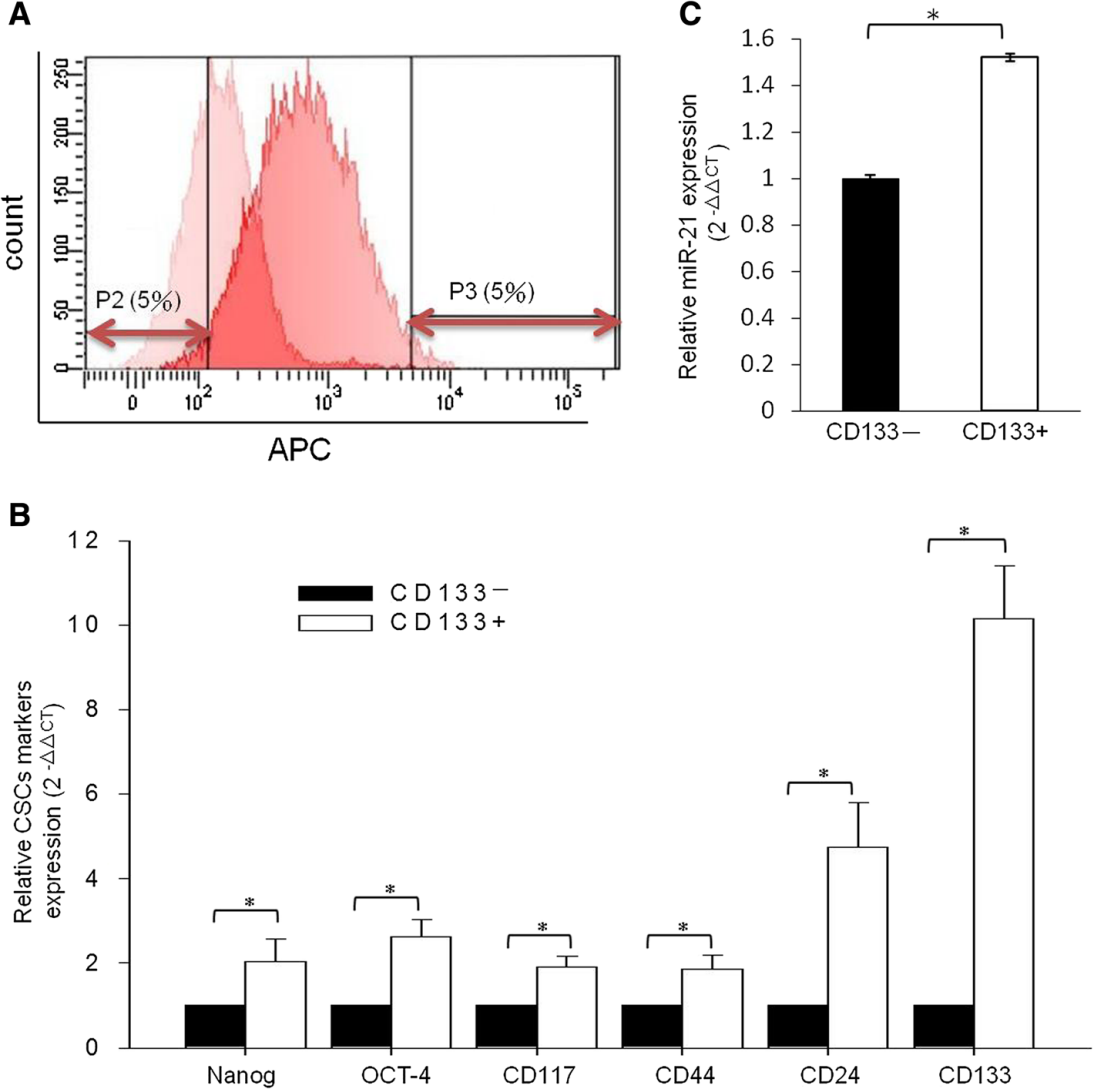
**MiR-21 was up-regulated in CD133+ PA1 cell populations.** APC-conjugated CD133 antibody was used to enrich CSPCs by FACS sorting. The basal IgG-isotype-APC staining signal peaks are presented on the left-hand side and the CD133-APC staining signal peaks are presented on the right-hand side of the histogram. As indicated in P2 and P3, the 5% extremes of the staining signal within the spectrum were sorted. Negatively stained cells (P2, left-hand side 5%) were defined as CD133- and positively stained cells (P3, right-hand side 5%) were defined as CD133+ cells **(A)**. CSPC marker genes were examined using quantitative real-time PCR, and the relative amount of each CSPC marker was calculated as 2^-ΔΔCt^. The expression of Nanog, OCT-4, CD117, CD44, CD24, and CD133 in CD133– cells was compared with that of each gene in CD133+ cells. **(B)**. MiR-21 expressed in CD133+ and CD133– cells. Quantitative real-time PCR was performed to detect expression of miR21. Expression of miR-21 in CD133– cells (basal level) was compared with the level of expression of miR-21 in CD133+ cells **(C)**. The data in **(B)** and **(C)** represent the mean of three individual sets of experiments. The variations were from the calculation of standard deviation, and * indicates statistical significance at *P*-values less than 0.05. APC, allophycocyanin; CSPCs, cancer stem/progenitor cells; FACS, fluorescence-activated cell sorting; IgG, immunoglobulin G; MiR, micro RNA.

CD133+ PA1 cells were sorted for further analysis. We gated the 5% extremes of the CD133 staining signal spectrum and defined them as CD133– (P2) and CD133+ (P3) cells (Figure 3A). To confirm that the collected cell population represented CSPCs, CD133 expression levels and other stem cell markers were examined using quantitative real-time PCR (qRT-PCR) (Figure 3B). We found that the expression of CD133 and other stem cell markers was higher in CD133+ cells than in CD133– cells, which suggests that isolated CD133+ cells exhibit CSPC characteristics. We also found that miR-21 levels were higher in CD133+ cells than in CD133– cells (Figure 3C). This finding indicates that miR-21 promotes PA1 cell growth by maintaining CD133+ CSPCs populations.

### MiR-21 promotes PA1 CSPC self-renewal

**Figure 4:**
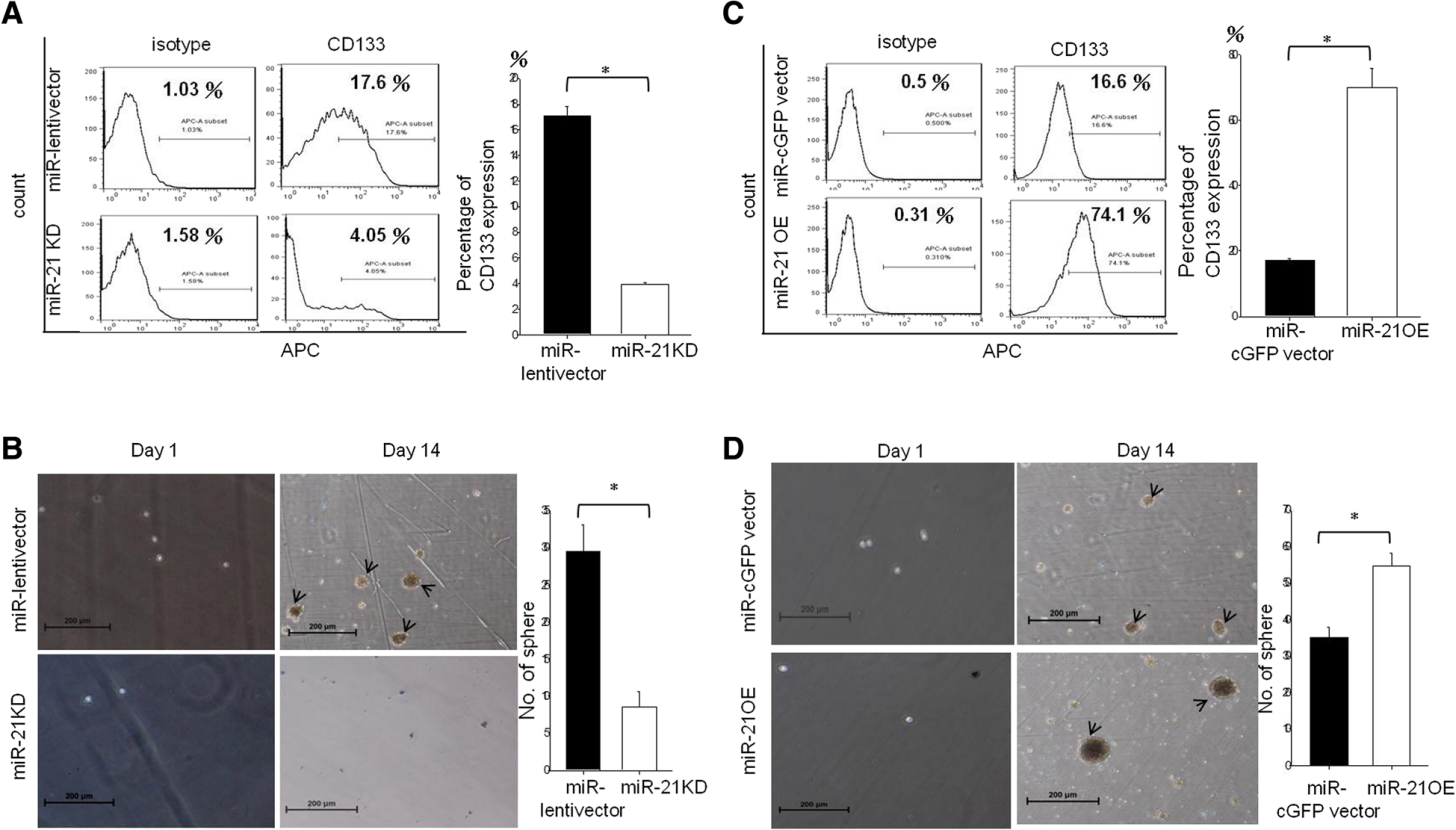
**MiR-21 is essential for PA1 CSPCs self-renewal.** Flow cytometric signal intensities were used to compare isotype-IgG-APC (left-hand side) and CD133-APC (right-hand side) in PA1 cells. Panel **A** shows the comparison of CD133+ populations in vector-infected cells (upper panel) and in anti-miR21-infected PA1 cells (lower panel). Sphere formation assay images of the PA1 cell colonies (black arrows indicate the tumor sphere). The sphere colony images were taken under a phase contrast fluorescence microscope (40×). The image on the left-hand side indicates vector-infected cells and the image on the right-hand side indicates anti-miR21-infected PA1 cell sphere colonies **(B)**. Signal intensities of isotype-IgG-APC in PA1 cells (left-hand side) were compared with those of CD133-APC in PA1 cells (right-hand side). CD133+ populations in vector-infected PA1 cells (upper panel) were compared with those in pre-miR21-infected PA1 cells (lower panel) **(C)**. Sphere formation assay images of the PA1 cell colonies (black arrows indicate the tumor sphere). The sphere colony images were taken using a phase contrast fluorescence microscope (40×). The image on the left-hand side indicates vector-infected cell sphere colonies and the image on the right-hand side indicates pre-miR21-infected PA1 cell sphere colonies **(D)**. The scale bar represents 200 μm. APC, allophycocyanin; CSPCs, cancer stem/progenitor cells; IgG, immunoglobulin G; miR, microRNA.

Functional assays were performed to confirm the effects of miR-21 on PA1 CSPCs. As seen in Figure 4A, miR-21 knockdown resulted in a marked reduction in CD133 populations compared with vector-infected PA1 cells (Figure 4A). We also found that knockdown of miR-21 resulted in little sphere formation compared with vector-infected controls after 14 days of culture (Figure 4B; black arrows indicate the tumor sphere). On the contrary, overexpression of miR-21 resulted in an increase in CD133 populations (Figure 4C) and CSPC sphere formation (Figure 4D; black arrows indicate the tumor sphere). Taken together, these data indicate that miR-21-related PA1 cell growth is mediated by CSPCs.

### MiR-21 is required for maintaining PA1 CD133+ CSPC

**Figure 5:**
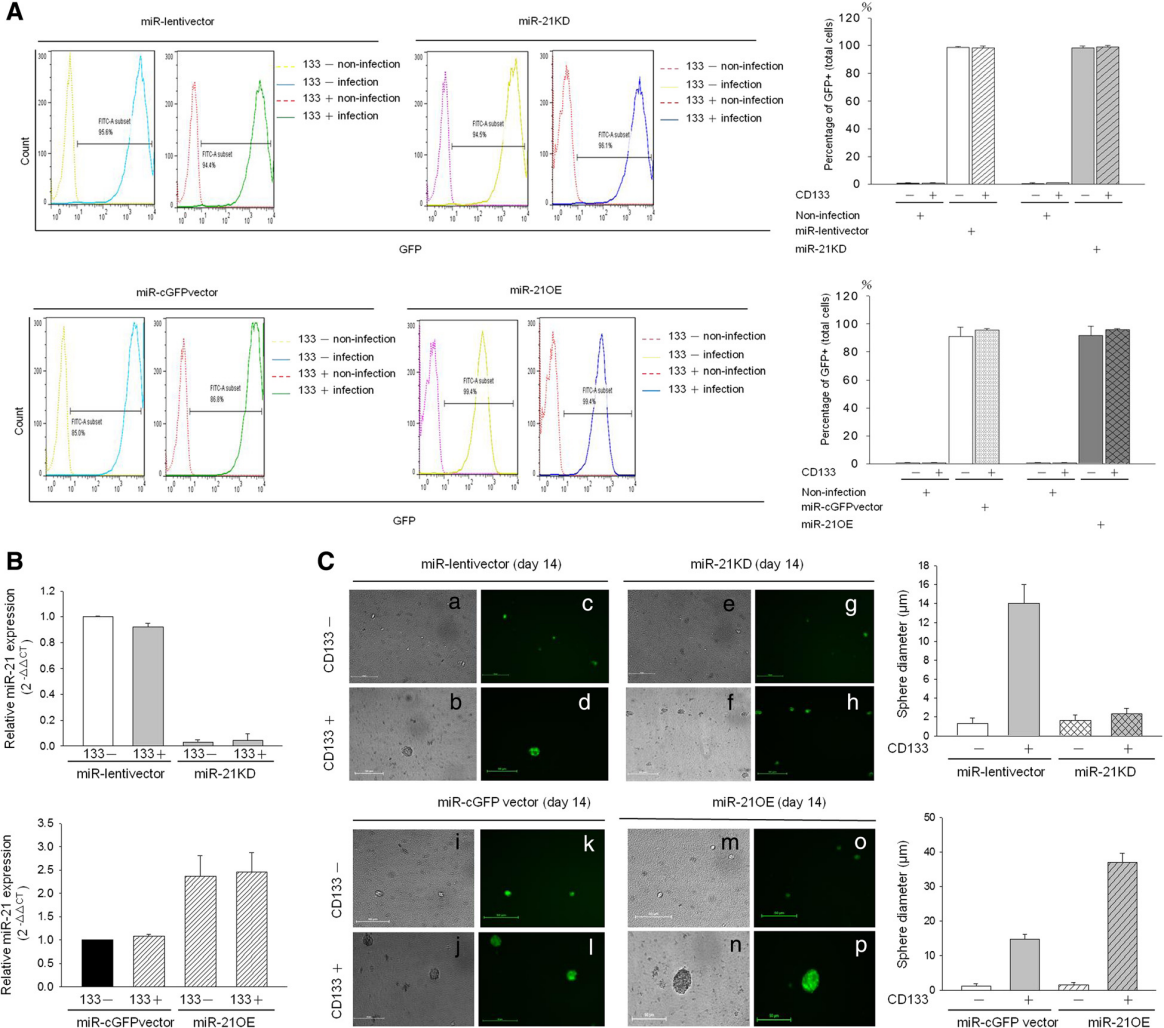
**MiR-21 is required for PA1 CD133+ CSPC function.** Purified CD133– and CD133+ populations from miR-lentivector, miR-21 KD, miR-cGFPvector and miR-21 OE were collected and the percentage of green fluorescence expression was detected by flow cytometry. The percentage of green fluorescence expression of infected cells was compared with that of non-infected cells. Flow cytometric signal intensities of non-infected cells were compared with those of infected cell populations (left-hand side). The percentages of infected cells with green fluorescence are presented on the right-hand side **(A)**. The relative expression of miR-21 in CD133+ and CD133– cells was detected using quantitative real-time PCR. Expression of miR-21 in CD133– cells (basal level) was compared with that of miR-21 expression in CD133+ cells **(B)**. Sphere formation assay images of the CD133+ and CD133– population colonies. The sphere colony images were taken under a phase contrast fluorescence microscope (100×). The images in the upper panel indicate vector-infected cells (left-hand side) and those on the right-hand side indicate anti-miR21-infected cells. The images of visible light and green fluorescence in the lower panel indicate vector-infected cells (left-hand side) and those on the right-hand side indicate miR21 overexpression **(C)**. The right panel represents quantitation of sphere diameters. Data in **(A)** and **(B)** represent the mean of three individual sets of experiments. The variations were from the calculation of standard deviation. The scale bar represents 50 μm. CSPC, cancer stem/progenitor cell; KD: knockdown miR, microRNA; OE.

To further prove that miR-21 is essential for CSPC growth and maintenance of its population, we evaluated sphere formation of CD133– and CD133+ cells that had been engineered to either underexpress or overexpress miR-21. CD133+/− cells were sorted using CD133 antibody-coated magnetic beads. The results of flow cytometry revealed that efficient gene transduction occurred in approximately 95% of PA1 cells (Figure 5A). We used qRT-PCR to detect expression of miR-21 in CD133– and CD133+ populations (Figure 5B). As seen in Figure 5B, miR-21 KD: knockdown (upper panel) led to a marked decrease in the expression of miR-21 and miR-21 OE: overexpression (lower panel) resulted in a marked increase in expression of miR-21. In order to test the hypothesis that miR-21 is essential for maintaining the CD133+ pluripotency of OVTC cells, we performed a sphere formation assay. We first used the lentiviral-delivery system to modulate miR-21 (Figure 5A and B) and the results revealed satisfactory infection efficiency. We then sorted CD133−/+ cells for the sphere formation assay (Figure 5C). As shown in Figure 5C, CD133+ cells formed more spheres than CD133– cells in the miR-lentivector- and miR-cGFP vector-infected groups (Figure 5C, left panel). Knockdown or overexpression of miR21 in CD133– cells exerted minor effects on PA1 sphere formation (first and third row), but suppressed (second row) or enhanced (fourth row) PA1 sphere formation in CD133+ cells, respectively. The quantified sphere sizes are shown on the right-hand side of Figure 5C.

Taken together, our results indicate that miR-21 is required for the maintenance of pluripotency of OVTC PA1 cells.

## Discussion

### MiR21 promotes cancer growth and progression

Studies have shown that miRNAs negatively regulate post-transcription in a variety of gene sets and are associated with proliferation, differentiation and progression in various cancers [1-7]. MiR-21 is up-regulated in different kinds of tumors, including breast and ovary [8-16]. Although several studies have indicated that miR-21 expression is high in CSPCs [6-11], the roles that miR-21 plays in CSPCs are still unclear. Herein we have shown that knockdown of miR-21 attenuated PA1 cell growth and that overexpression of miR-21 promoted cell growth.

A number of studies have shown that CD133 is a marker of cancer stem/progenitor cells [30,40,41], and functional assays of CSPCs have revealed that enriched CD133 subpopulations from ovarian cancer tissue are indeed CSPCs [30,40-44]. By manipulating miR-21 levels in the cells, we found that miR-21 is required for maintenance of CD133 populations. Although this finding is consistent with that reported in a previous study [8], it has never been shown in OVTC cells.

Tumor cells with CSPC characteristics grown in suspended conditions with serum-free stem cell culture medium can form tumor spheres [27,29]. The sphere formation assay is widely used to evaluate the self-renewal ability of cancer stem cells *in vitro *[19,27,28]. In addition to testing the effects of miR-21 on characteristics of cancer progenitor cells, we also examined the self-renewal of CSPCs by conducting a sphere formation assay and found that miR-21 suppresses PA1 cell growth by maintaining CD133+ populations.

### Chemotherapy resistance might be associated with miR21-related CSPCs

Chemotherapy resistance and cancer recurrence are major obstacles in the treatment of cancer. Various miRs are associated with cancer cell proliferation and drug resistance [41]. Expression of miR-21, for example, has been demonstrated to be related to chemoresistance in glioma [45], breast [46,47], bladder [48] and neuroblastoma cancer cells [49]. MiR-21 has also been shown to be linked to resistance to at least nine chemotherapy agents, for example, temozolomide [41], trastuzumab [45,46], doxorubicin [47], cisplatin [48], Triptolide [50], and CHOP (cyclophosphamide, vincristine, Adriamycin,and prednisone) [51]. Our finding that miR-21 enhances cancer stemness might explain why this small RNA molecule is responsible for resistance to so many chemotherapeutic agents. In our study, knockdown of miR-21 expression suppressed cell growth and tumor cell sphere formation. Moreover, we demonstrated that miR-21 is up-regulated in CD133+ cells (Figure 3C), indicating that it might be important for the maintenance of CSPC populations. CD133+ cells have been reported to be associated with drug resistance in glioblastoma [52], Ewing sarcoma cells [53], lung cancer [54,55] and hepatocellular carcinoma [56]. Our findings indicate that miR-21 plays a critical role in regulating CD133+ CSPCs.

## Conclusions

Our study revealed that miR-21 regulates CSPCs populations in PA1 cells. MiR-21, therefore, is a potential target for cancer therapy, especially in patients with cancer relapse.

## Abbreviations

BSA: bovine serum albumin; CSPCs: cancer stem/progenitor cells; Ct: threshold value; (D)MEM: (Dulbecco’s) modified Eagle’s medium; EDTA: ethylenediaminetetraacetic acid; ELISA: enyme-assisted immunosorbent assay; FACS: fluorescence-activated cell sorting; FBS: fetal bovine serum; FCS: fetal calf serum; GFP: green fluorescent protein; hrbFGF: human recombinant basic fibroblast growth factor; hrEGF: human recombinant epidermal growth factor; MACS: magnetic cell separation; miR-21: microRNA-21; OVTC: ovarian teratocarcinoma; PBS: phosphate-buffered saline; PCR: polymerase chain reaction; UTR: untranslated region.

## Competing interests

The authors declare that they have no competing interests.

## Authors’ contributions

WMC and WCC executed the major experiments and drafted the manuscript. LMC and YYC helped with the design of experiments and manuscript preparation. CRS provided insightful criticism and suggestions on the manuscript. YCH supported and helped with manuscript preparation. WLM supported and supervised the overall project and final approval of the manuscript. All authors read and approved the final manuscript.
